# Joint Transcriptomic Analysis of Lung Cancer and Other Lung Diseases

**DOI:** 10.3389/fgene.2019.01260

**Published:** 2019-12-06

**Authors:** Beatriz Andrea Otálora-Otálora, Mauro Florez, Liliana López-Kleine, Alejandra Canas Arboleda, Diana Marcela Grajales Urrego, Adriana Rojas

**Affiliations:** ^1^Facultad de Medicina, Universidad Nacional de Colombia, Bogotá, Colombia; ^2^Departamento de Estadística, Grupo de Investigación en Bioinformática y Biología de sistemas – GiBBS, Facultad de Ciencias, Universidad Nacional de Colombia, Bogotá, Colombia; ^3^Departamento de Medicina Interna, Hospital Universitario San Ignacio, Bogotá, Colombia; ^4^Instituto de Genética Humana, Facultad de Medicina, Pontificia Universidad Javeriana, Bogotá, Colombia

**Keywords:** lung cancer, interstitial lung diseases (ILDs), pulmonary arterial hypertension (PAH), differentially expressed genes (DEGs), coexpression networks, early detection and prognosis biomarkers, survival

## Abstract

**Background:** Epidemiological and clinical evidence points cancer comorbidity with pulmonary chronic disease. The acquisition of some hallmarks of cancer by cells affected with lung pathologies as a cell adaptive mechanism to a shear stress, suggests that could be associated with the establishment of tumoral processes.

**Objective:** To propose a bioinformatic pipeline for the identification of all deregulated genes and the transcriptional regulators (TFs) that are coexpressed during lung cancer establishment, and therefore could be important for the acquisition of the hallmarks of cancer.

**Methods:** Ten microarray datasets (six of lung cancer, four of lung diseases) comparing normal and diseases-related lung tissue were selected to identify hub differentiated expressed genes (DEGs) in common between lung pathologies and lung cancer, along with transcriptional regulators through the utilization of specialized libraries from R language. DAVID bioinformatics tool for gene enrichment analyses was used to identify genes with experimental evidence associated to tumoral processes and signaling pathways. Coexpression networks of DEGs and TFs in lung cancer establishment were created with Coexnet library, and a survival analysis of the main hub genes was made.

**Results:** Two hundred ten DEGs were identified in common between lung cancer and other lung diseases related to the acquisition of tumoral characteristics, which are coexpressed in a lung cancer network with TFs, suggesting that could be related to the establishment of the tumoral pathology in lung. The comparison of the coexpression networks of lung cancer and other lung diseases allowed the identification of common connectivity patterns (CCPs) with DEGs and TFs correlated to important tumoral processes and signaling pathways, that haven´t been studied to experimentally validate their role in the early stages of lung cancer. Some of the TFs identified showed a correlation between its expression levels and the survival of lung cancer patients.

**Conclusion:** Our findings indicate that lung diseases share genes with lung cancer which are coexpressed in lung cancer, and might be able to explain the epidemiological observations that point to direct and inverse comorbid associations between some chronic lung diseases and lung cancer and represent a complex transcriptomic scenario.

## Introduction

Lung cancer is the leading cause of cancer death in men and women in the world (Siegel et al., 2015). Lung cancer is a malignant tumor of uncontrolled cell growth with the ability to metastasize in distant tissues (Dela Cruz et al., 2011). Lung cancer can be divided into two main histological types: non-small cell cancer (NSCLC), representing 85% of all lung cancers, and small cell cancer (SCLC) (Gridelli et al., 2015). The global cancer burden reported by GLOBOCAN 2018 and the International Agency for Research on Cancer estimates that there will be 18.1 million new cancer cases (Bray et al., 2018). Lung cancer is the most commonly diagnosed cancer (11.6% of total cases) and the leading cause of cancer death (18.4% of total deaths due to cancer), followed by breast cancer (11.6%), prostate cancer (7.1%) and colorectal cancer (6.1%) (Bray et al., 2018). Lung cancer is the most frequent cancer and the leading cause of cancer death in men. In women, breast cancer is the most frequently diagnosed cancer and the leading cause of cancer death, followed by colorectal and pulmonary cancer (IARC and Boyle, 2018). The increase in mortality rates during the last decades have been associated with a late diagnosis, which limits their potential treatment, and is evidence of the important lack of biomarkers for the development of specific treatments against the disease (Dela Cruz et al., 2011).

The interstitial lung diseases (ILDs) are a heterogenic group of conditions with clinic, radiologic, and functional manifestations, among which the most important are anatomopathological alterations that affect alveolointerstitial structures, like the epithelium, alveolar walls, capillary endothelium and connective tissue (perilymphatic and perivascular) between the septa and the peribronchial and peribronchiolar tissue (Raghu et al., 2018). Environmental factors (silica dust, asbestos fibers, grain dust, bird droppings, and animals), infections, medications (anti-inflammatory, antibiotics and cardiovascular), radiation exposure, and autoimmune, granulomatous, metabolic, systemic (connective tissue) and childhood-specific diseases are between the known associated causes (Raghu et al., 2004; Nathan et al., 2012; Griese et al., 2015). The ILDs can affect men and women of any age who are exposed to specific concentrations of any of the environmental factors, and/or are carriers of genetic and epigenetic factors that predispose them for the development of the disease (Coultas et al., 1994; Griese et al., 2015). The ILDs include a wide range of diffuse pulmonary disorders that often end in pulmonary fibrosis and may occur in isolation or associated with systemic diseases (Spagnolo et al., 2014; Kropski et al., 2015).

In recent decades, studies have shown a possible relationship between pathological processes in lung tissue and the development of cancer. Specifically, ILDs and lung cancer share pathogenic mechanisms such as inflammation, increased resistance to apoptosis, focal hypoxia, an increase in proliferation, viability and accumulation of cells such as fibroblasts, in regions with repeated epithelial lesions (Archontogeorgis et al., 2012). The epithelial mesenchymal transition (EMT) process characteristic of tumor cells and essential to carrying out metastasis processes, is a phenomenon that also occurs in epithelial type II alveolar cells that are transformed into mesenchymal cells to produce fibroblasts and myofibroblasts that directly contribute to the fibrotic event in ILDs (Willis and Borok, 2009). In lung diseases, epithelial cells exposure to matrix metalloproteinases can lead to an increase in reactive oxygen species levels that promote myofibroblasts differentiation (Sokai et al., 2015). The deregulation of metalloproteinases expression associated with a defective matrix and increase levels of reactive oxygen species are also characteristic of malignancy, suggesting a relationship between the two pathologies (Radisky et al., 2007). Several studies suggest a possible association between other lungs diseases and lung cancer, through different physiopathogenic mechanisms. The presence of common tumorigenic processes between lung cancer and other lung diseases suggests a possible causal association in the establishment of this tumoral pathology, and that the joint transcriptomic analysis can ultimately result in identifying potential novel biomarkers/drug targets for lung cancer early diagnosis, if it is associated with the risk.

Cancer Biomarkers are used in clinical practice for diagnosis, prognosis, the identification of sensitive patients, and the prediction of cancer patients response to treatment (Kamel and Al-Amodi, 2017). There are several types of biomarkers: Prognostic biomarkers are used to identify the patients that need treatment in the future, Predictive biomarkers direct patient’s treatment selection, and Diagnostic biomarkers contribute to the diagnosis and classification of the disease, and can be useful to monitor the therapeutic response in patients (Fenton et al., 2011). Cancer research has identified a significant number of predictive biomarkers for some types of cancer according to their clinical usefulness (Kamel and Al-Amodi, 2017). In lung cancer NSCLC have been identified some predictive biomarkers whose expression can predict the response to a specific treatment (BRCA1, TP53, and KRAS) (Kamel and Al-Amodi, 2017).

The identification of genetic risk factors, as mutations in somatic and germ cells (*EGFR*, *TP53*, *KRAS*, *BRAF*, *ERBB2*, *MET*, *STK11*, *PIK3CA*), gene amplifications (*EGFR*, *ERBB2*, *MET*, *PIK3CA*, and *NKX2*), deletions (DOK2) (Berger et al., 2010), and presence of fusion genes (*ALK/EML4*) (Soda et al., 2007), have allowed to develop specific treatments, but although they increase the susceptibility to develop lung cancer (Braun et al., 1994), they have not shown a concordance with the mortality rates because their limited ability to treat patients only with the associated risk factor (Blanchon et al., 2006). In the postgenomic era, the biomarker identification studies have begun to take into account two important components for clarifying the etiology of the tumoral diseases: First, the great inter and intratumoral variability at the molecular and cellular level in each individual and/or population of individuals; and second, the complexity of lung cancer, in terms of the huge number of deregulated genes that have been identified with genomic studies, which highlights the complexity of cancer and generates a problem in the development of therapeutic treatments for each possible variation, that must be solved.

Genomic studies such as microarrays and RNA-Seq have the ability to provide information for the identification of all groups of transcriptionally dysregulated genes involved in the modulation of biological functions and signaling pathways, describing expression patterns associated with tumor grade, differentiation state, metastatic potential, and patients’ survival, when comparing tumoral and healthy tissue (Wang and Liotta, 2011; Barretina et al., 2012; Eswaran et al., 2013; Li et al., 2017; Ma et al., 2019). Our research group believes that the combined bioinformatics analysis of a selected group of these studies can take advantage of all the transcriptomic knowledge generated with these technologies, finding potentially valuable information that could be applied to increase the understanding of cellular processes related to complex diseases such as cancer. This kind of analysis can help us to understand the complexity of the disease from a global perspective, through the identification of all deregulated genes that participate in the establishment of lung cancer. Additionally, the comparison of different pathologies when performing a global analysis of several databases, allow us to find specificities and common processes, which, when analyzing each study separately, cannot be detected.

The study of complex systems can be based on communication between signaling pathways in gene networks, and on the identification of prognosis modules in co-expression networks associated with a biological process, which can become good targets for pharmaceutical treatment. (Zhou et al., 2019). Currently, published studies look for cancer biomarkers associated with the hallmarks of cancer (Hanahan and Weinberg, 2011), based on the generation of co-expression networks using transcriptomic studies in different types of cancer (Yu et al., 2019b), through the weighted gene coexpression networks (WGCNA) method, which identified more than 3,000 coexpression modules associated with tumoral processes such as cell proliferation, extracellular matrix remodeling, hypoxia, inflammation, angiogenesis, and cell differentiation (Ivliev et al., 2016); with Gaussian mixing models (GMM) an important number of modules in a pairwise gene comparison to reduce extrinsic background noise in the co-expression network (Ficklin et al., 2017); and using a permutations test, the communities present in all networks have been identified, followed by an enrichment analysis to identify the associated biological processes, and a survival analysis to differentiate communities capable of differentiating cancer patients from healthy individuals (Yu et al., 2019a).

We propose a new methodology for the creation of co-expression networks, which was developed by Dr. López-Kleine research group, based on the use of the library “Coexnet” (Henao et al., 2018). This method allows the creation of co-expression networks from microarray data sets, the identification of multiple nodes connected in an intersection network in common when comparing two or several biological networks, which have been called common connectivity patterns (CCPs) (Henao et al., 2018). Then, Coexnet allows to create coexpresión networks of lung cancer and other lung diseases, to compare them and identify CCPs in common between pathologies, and TFs deregulated and coexpressed during the establishment of tumoral pathology in the lung. Therefore, we were able to visualize the complexity of lung cancer, as the previous methods, however, we also generate within the same bioinformatic pipeline a methodology to address the huge complexity of the disease, identifying the transcriptional regulators of all deregulated and coexpressed genes in lung cancer associated with the acquisition of the hallmarks of cancers, in order to take a reasonable number of genes as candidate biomarkers into the experimental validation phase, for the future the development of specific and personalized treatments of lung cancer.

## Methods

### Selection of Gene Expression Data

Ten sets of expression data (microarrays) were selected from the public repositories Gene Expression Omnibus (GEO) from NCBI and ArrayExpress from EBI. Six sets compare tumor tissue from patients with lung cancer and normal lung tissue, and the other four sets compare lung tissue affected with different lung diseases with normal lung tissue (**Table 1**). The analysis follows 3 stages: 1) Data Pre-processing (quality verification, filter, normalization, etc.) based on the methodology proposed by Leal et al. (2014) 2) Detection of differentially expressed genes (DEGs) (Oshlack et al., 2010), 3) Identification of transcription factors among DEGs detected. In the first stage, the quality of the data was evaluated in order to work with those that have acceptable experimental validity. In the second and third stage, an important number of DEGs associated with biological functions relevant to the oncogenic process were obtained from other lung diseases and lung cancer.

**Table 1:** Analyzed data sets, each study code, subjects’ related disease, number of samples per condition and reference.

Study code	Subjects disease	Number of samples	Reference
**GSE19804**	Non-smoking women with NSCLC	Normal (60) vs. Cancer (60)	(Lu et al., 2010)
**GSE10072**	Patients with lung adenocarcinoma	Normal (49) vs. Cancer (58)	(Landi et al., 2008)
**GSE3268**	Patients with Squamous lung cancer cells	Normal (5) vs. Cancer (5)	(Wachi et al., 2005)
**GSE108055**	Typical and atypical carcinoid, and small cell lung cancer	Normal (9) vs. Cancer (54)	(Asiedu et al., 2018)
**E-MTAB-5231**	Patients with NSCLC	Normal (18) vs. Cancer (22)	(Willuda et al., 2017)
**E-MTAB-3950**	Pre-invasive and Invasive Early Squamous Carcinoma	Normal (30) vs. Cancer (30)	(Koper et al., 2017)
**GSE21411**	Interstitial lung diseases (ILDs)	Normal (12) vs. ILD (42)	(Cho et al., 2011)
**GSE1650**	Emphysema	Normal (12) vs. Emphysema (18)	(Spira et al., 2004)
**GSE2052**	Idiopathic pulmonary fibrosis (IPF)	Normal (11) vs. IPF (13)	(Pardo et al., 2005; Kim et al., 2006)
**GSE113439**	Pulmonary arterial hypertension (PAH)	Normal (11) vs. PAH (15)	None publications in PubMed

### Differentially Expressed Genes (DEGs) Analysis

Differentially expressed genes (DEGs) identification is based on finding if there is statistical evidence to declare that a gene is more or less active in a condition (“lung pathology”) with respect to a control (“normal lung”). R language (R Core Team, 2019) and specialized libraries were used to identify DEGs in every selected data set. First, pre-processing and quality control of each data set was performed through exploratory analysis of the data. This analysis consisted of evaluating the similar gene expression distribution per sample and examining that there were no genes with atypical expressions or samples that had a different behavior with respect to the others, through boxplots, density plots and summary statistics. Second, normalization was conducted in each data set in order to reduce data dispersion and make samples comparable each other using Variance Stabilizing Normalization (VSN) method using VSN library (Huber et al., 2002; Gautier et al., 2004), evaluating the similar gene expression distribution per sample in boxplots and density plots to use only statistically comparable data sets. When each data set passed pre-processing and normalization steps, it proceeded with the detection of DEGs.

The DEGs were selected based on the method of Significance analysis of microarray data (SAM) employing SAM function of siggenes package (Schwender, 2012), with the lowest error rate (q-value < 0.01), and under 15% FDR (Tibshirani et al., 2018). The q-value (Storey’s q-value) indicates the probability that a DEG identified is a false positive, corrected by multiple tests significance. This analysis was carried out for both lung cancer (LC) and other lung diseases (LD) data sets and the DEGs list in each data set was divided into positive and negative deregulated genes according to its fold change. Finally, all common genes between LC and other LD, varying in the same sense (over-expressed or repressed in the pathology with respect to healthy tissue) in the majority, 7 of the 10 sets analyzed, (in at least five of the six lung cancer sets, and one or two of other lung disease) were established as our “winner DEGs”. These were highlighted in the functional analysis, classifying those involved in tumorigenic processes.

### Functional Annotation and Enrichment Analysis

The analysis of enrichment and functional annotation allows the identification of significant and specific biologic functional categories within our list of genes, considering the current scientific knowledge and the organism. This analysis was carried out with the online DAVID tool (Bioinformatics Resources 6.8, NIAID/NIH” (https://david.ncifcrf.gov/summary.jsp) (Dennis et al., 2003), which associates the winner DEGs found with specific biological functions. The analysis of associated diseases and functional categories for winning genes positively or negatively deregulated in the majority of sets of lung cancer and in other lung diseases, allow us to identify the DEGs with greater scientific evidence associated with oncogenic processes. The P value was corrected (because of multiple tests analyses) with Benjamini’s method (Benjamini and Hochberg, 1995), and it was considered a significant enrichment for functional categories P values ≤0.05. An extensive literature search in the common overregulated winner DEGs related to cancer was preformed to validated its association with lung cancer and other lung diseases.

### Coexpression Network Analysis

The web-based system oPOSSUM (http://opossum.cisreg.ca/cgi-bin/oPOSSUM3/opossum_human_ssa) was used to detect over-represented conserved transcription factor binding sites and binding site combinations in the 45 overexpressed genes related to cancer according to DAVIDs analysis and verified with literature searching. The common 45 DEGs and the 45 TF (90 genes in total) were used to construct a gene co-expression network, a heatmap and a corrplot. The normalized expression profiles of the 59 of the 77 genes of interest were extracted from the gene expression data set GSE19804 and a similarity matrix was constructed using the absolute value of Pearson correlation. On this matrix, the threshold was identified to establish a final edge on the co-expression network using the methodology of Leal et al. (2014), based on the comparison of the obtained clustering coefficient with the clustering coefficient of a random graph (Leal et al., 2014).

The R library “Coexnet” was used to construct coexpression networks of one lung cancer data set (GSE19804) and the four sets of other lung diseases (GSE21411, GSE1650, GSE2052, GSE113439). Coexnet compared each lung disease network with the lung cancer network in order to identify common connectivity patterns (CCPs) between them, allowing the identification of molecular components linked together and common in the biological networks (Henao et al., 2018). CCPs networks analysis with Cytoscape (Shannon et al., 2003) and iRegulon application allowed the identification of transcription factors or master regulators of every CCP identified (Janky et al., 2014).

### Survival Analysis of Transcription Factors

The effect of the expression of the hub genes or TFs identified with the transcriptomic, enrichment and coexpression analyses, in the survival of patients with lung cancer was assessed with the Kaplan Meier-plotter tool (KM plotter: http://kmplot.com/analysis/index.php?p=service&cancer=lung) (Gyorffy et al., 2013). KM plotter information about overall surviving (OS) is calculated and plotted with datasets from GEO, EGA and TCGA, based on hazard ratio (HR) of 95% confidence intervals and with a log rank P-value (LogrankP-value) associated.

## Results

### Differentially Expressed Genes (DEGs) in Common Between Lung Cancer and Other Lung Diseases

**Figure 1:**
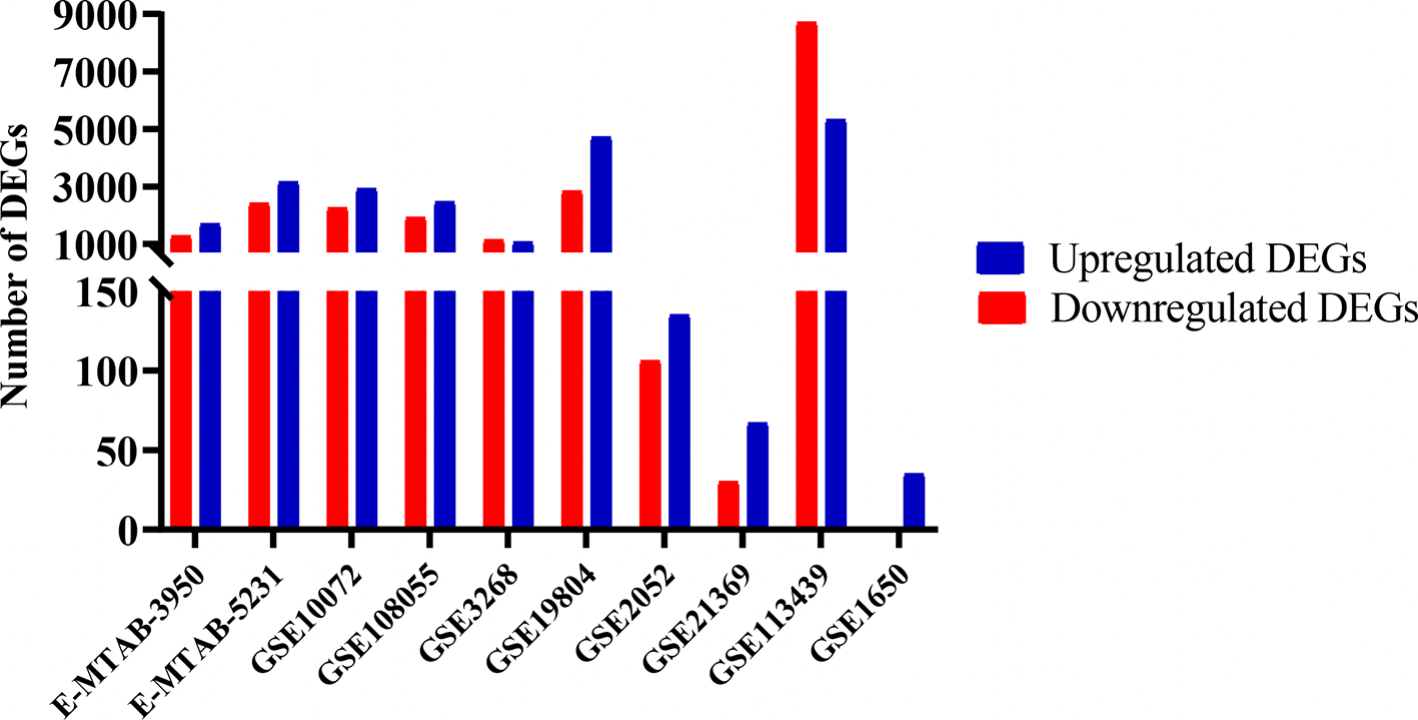
Differentially expressed genes (DEGs) in lung cancer and other lung diseases studies. The image was made using GraphPad Prism version 8.00 for Windows, GraphPad Software, La Jolla California USA, www.graphpad.com.

In general, there is a greater number of DEGs in lung cancer than in other lung diseases, and a greater number of overregulated genes than downregulated genes in both groups (**Figure 1**). However, there is a high number of deregulated genes in Pulmonary arterial hypertension (PAH) dataset equivalent to lung cancer datasets. The identification of 395 genes that were differentially expressed in lung cancer and other lung diseases in at least seven sets of the 10 data sets analyzed, indicated that the joint microarray data sets analysis allows us to find a characteristic metafirm of lung diseases establishment processes at molecular level. 210 of the DEGs were equally deregulated in both, lung cancer and other lung diseases, among which 116 were overregulated and 94 downregulated (**Supplementary Table 1**).

### Functional Annotation and Enrichment Analysis

**Figure 2:**
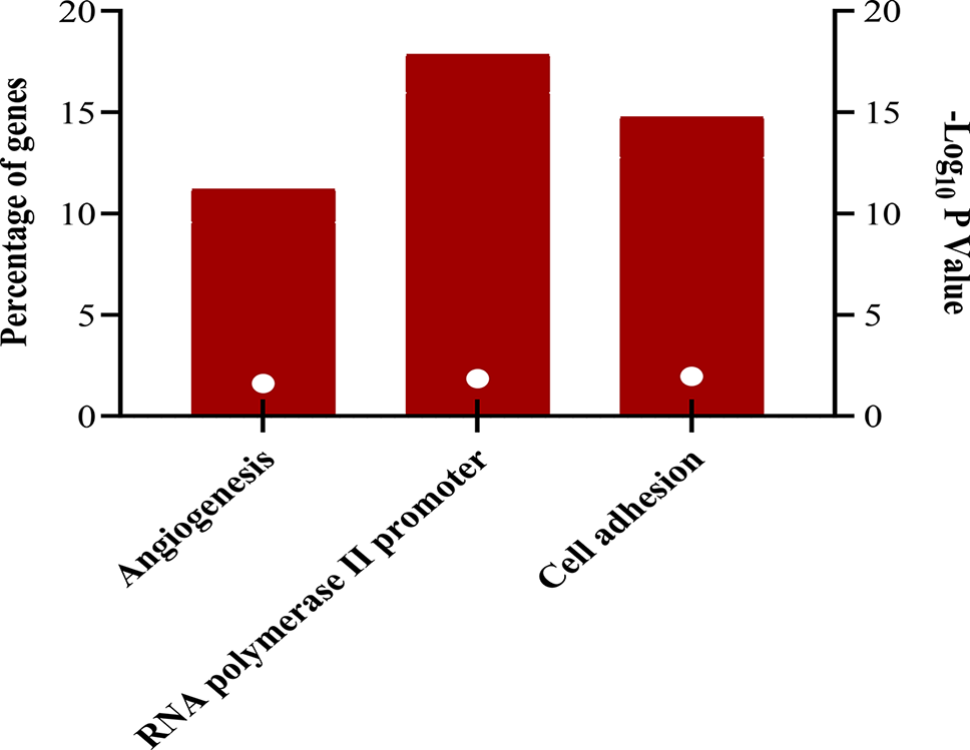
Biological processes associated to downregulated genes common in lung cancer and other lung diseases. The image was made using GraphPad Prism version 8.00 for Windows, GraphPad Software, La Jolla California USA, www.graphpad.com.

**Figure 3:**
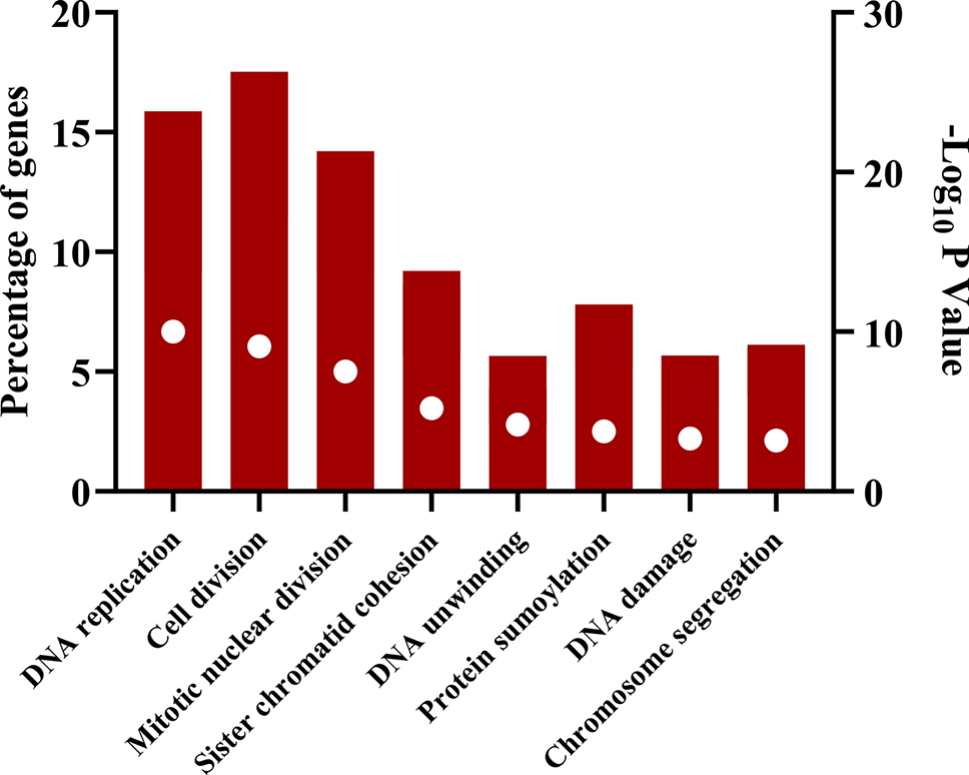
Biological processes associated with common overregulated genes in lung cancer and other lung diseases. The image was made using GraphPad Prism version 8.00 for Windows, GraphPad Software, La Jolla California USA, www.graphpad.com.

**Figure 4:**
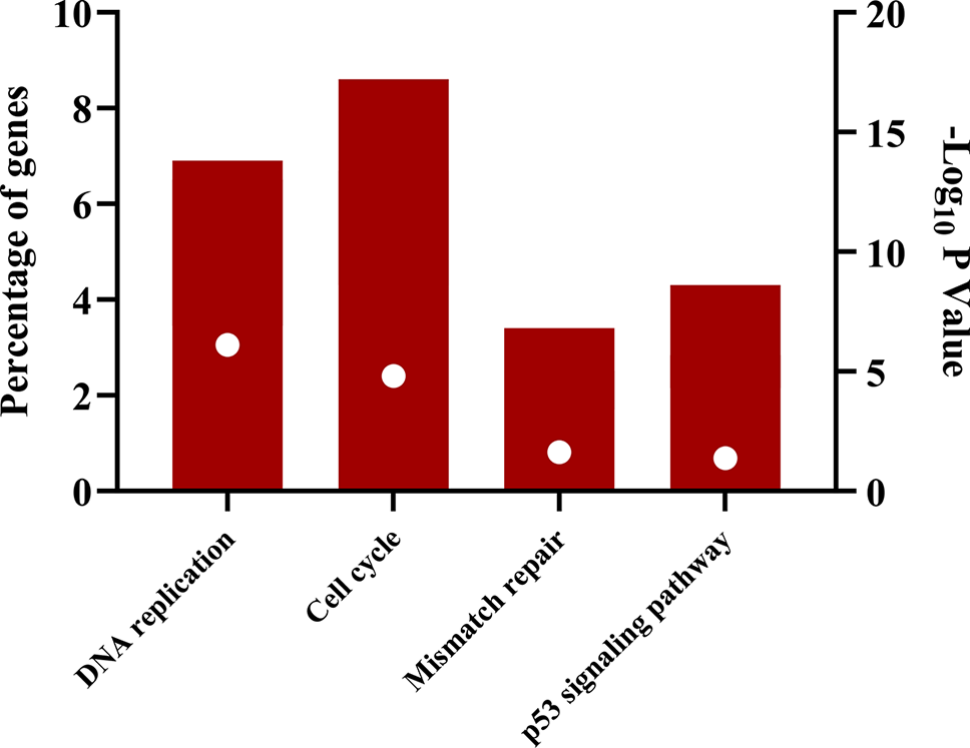
Signaling pathways associated with common overregulated genes in lung cancer and other lung diseases. The image was made using GraphPad Prism version 8.00 for Windows, GraphPad Software, La Jolla California USA, www.graphpad.com.

The downregulated “winner” DEGs are associated with angiogenesis, cell adhesion and the negative regulation of transcription from RNA polymerase II promoter (**Figure 2**). The overregulated “winner” DEGs are associated with several cell cycle processes (**Figure 3**), DNA replication, mismatch repair, and p53 signaling pathways (**Figure 4**). The functional annotation analysis of the 116 overregulated common genes identified 45 genes associated with cancer with a significant corrected P value.

### Coexpression Network Analysis

**Figure 5:**
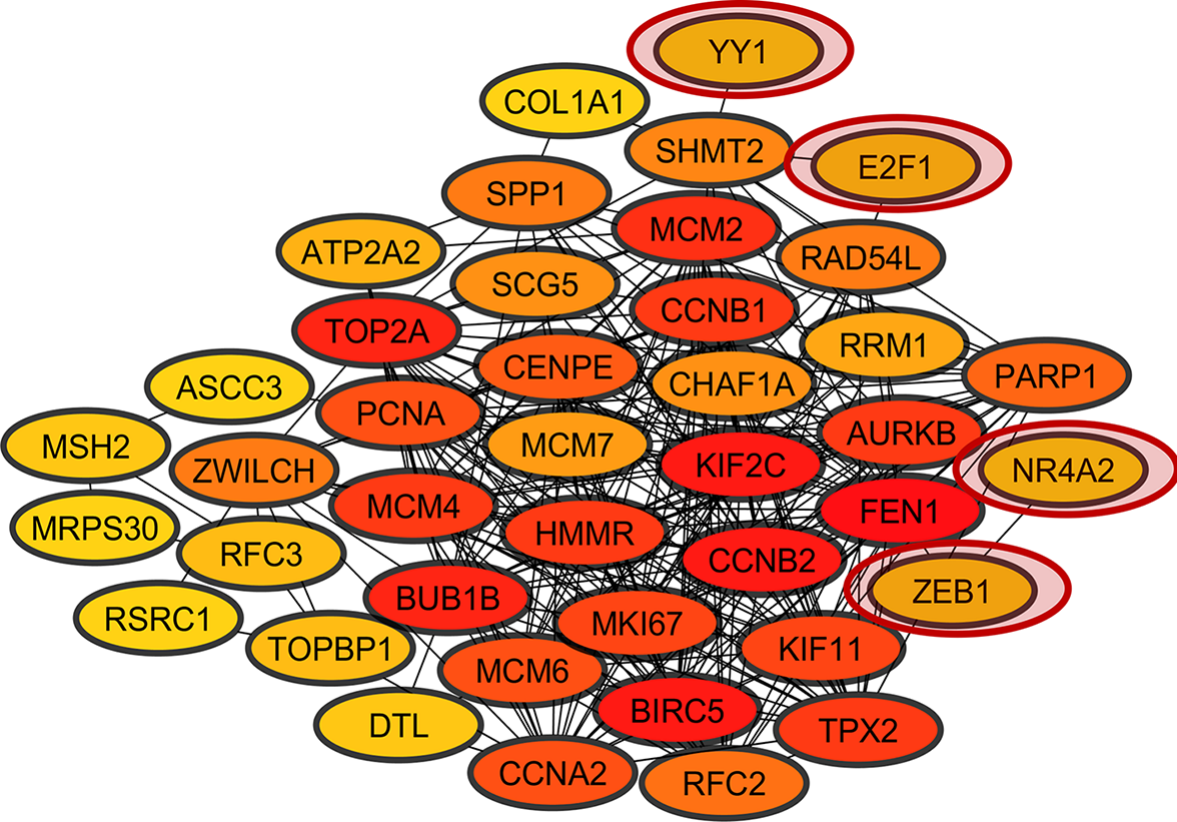
Coexpression network of overregulated genes common in lung cancer and other lung diseases (LC&LD coexpression network). The red circles show the TFs coexpressed in the network.

**Figure 6:**
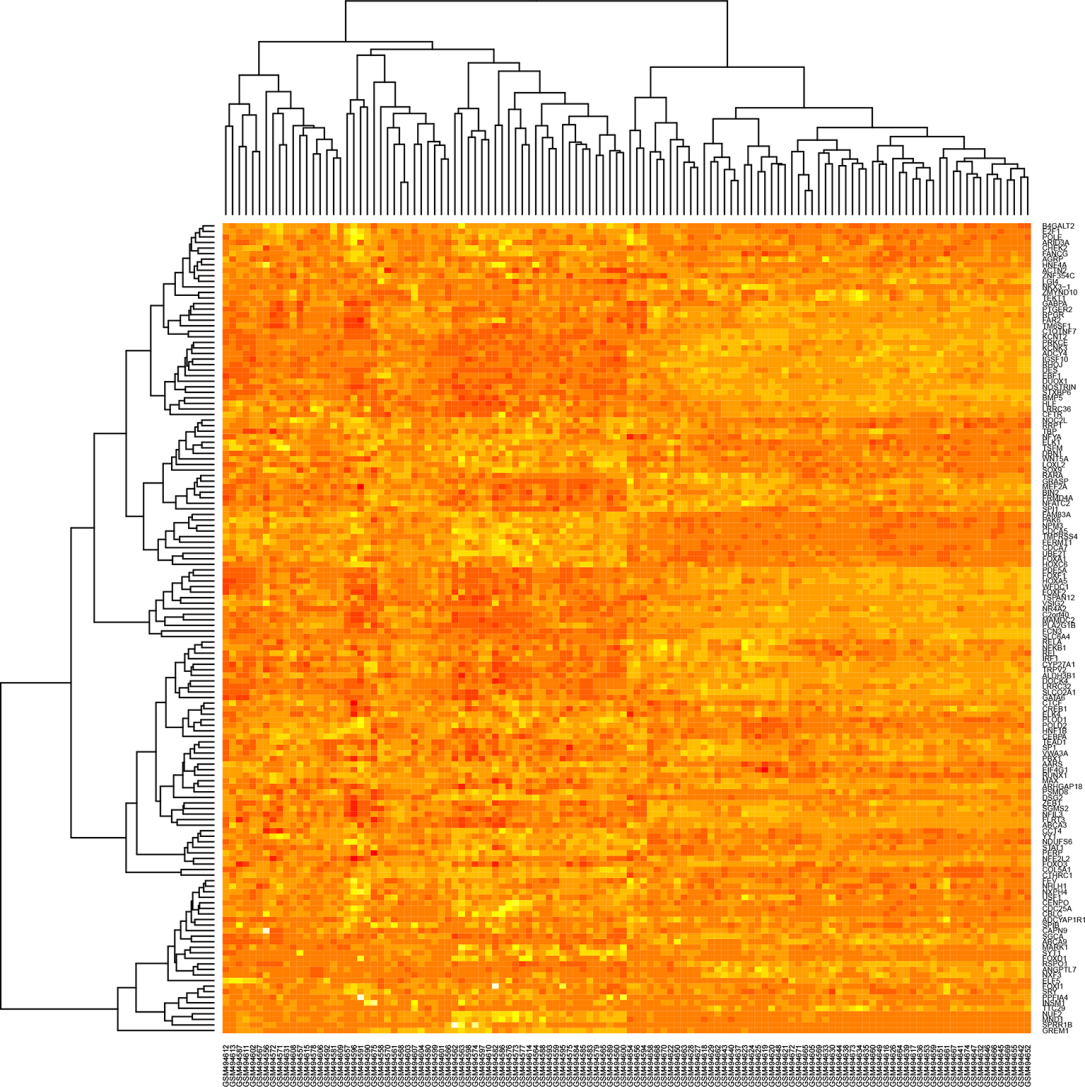
Heatmap of the winner deregulated genes common in lung cancer and other lung diseases.

**Figure 7:**
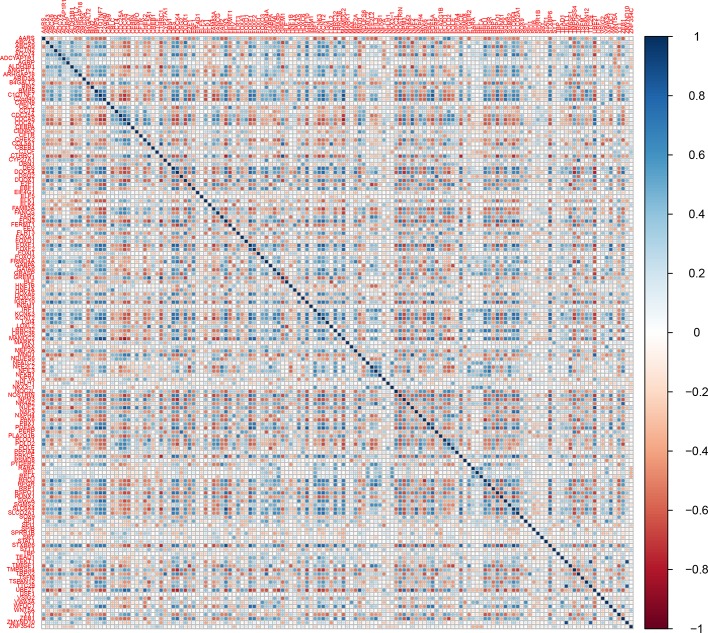
Corrplot of the winner deregulated genes common in lung cancer and other lung diseases.

The heatmap and corrplot show The coexpression network created with the 45 DEGs associated with cancer in DAVIDs enrichment analysis and the 45 TFs identified with Opossum as their possible regulators, has 35 DEGs coexpressed with four TFs (YY1, ZEB1, E2F1, and NR4A2) (**Figure 5**) (Shannon et al., 2003). The heatmap and corrplot show the correlation between all the genes used to create the network (**Figures 6** and **7**). YY1 is overregulated in three lung cancer datasets and downregulated in PAH dataset. NR4A2 is downregulated in five lung cancer datasets and overregulated in PAH dataset. ZEB1 is downregulated in four lung cancer and ILDs datasets, and overregulated in PAH. E2F1 is only overexpressed in five lung cancer datasets. The extensive literature search associates most of the coexpression network genes with lung cancer, and most of them with other lung diseases (**Supplementary Table 2**). 36 genes of the coexpression network were deregulated in PAH, 12 genes in IPF, 1 in ILDs, and 1 in emphysema dataset, but none was reported or analyzed in their specific lung disease study.

**Figure 8:**
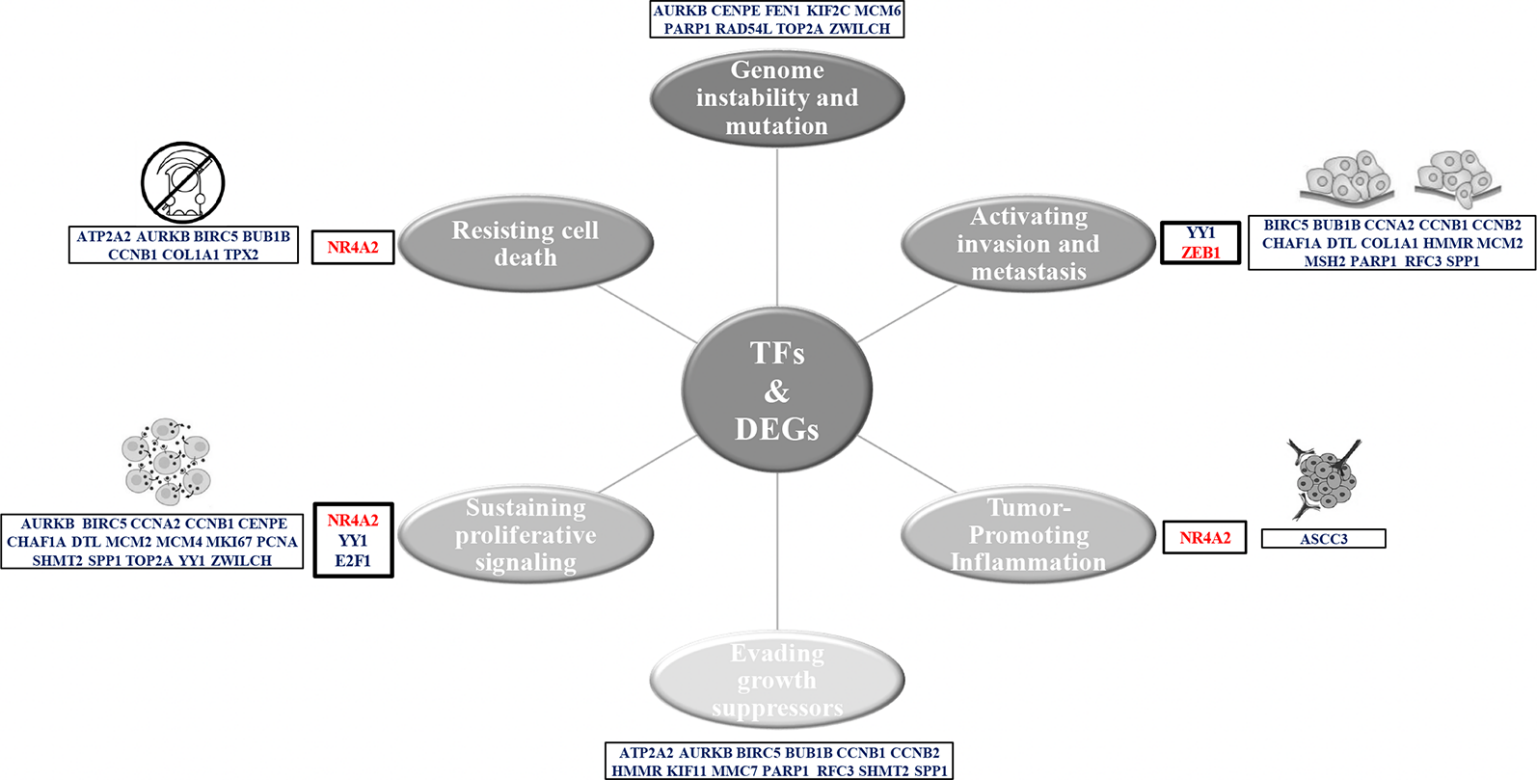
Hallmarks of cancer related to the genes of the LC&LD coexpression network.

Furthermore, the coexpression network genes were related with the seven of the 10 Hallmarks of Cancer proposed by Hanahan and Weinberg (2011) (**Figure 8**). NR4A2 and E2F1 function have been related with resisting to cell death and sustaining a proliferative signal processes in lung cancer. Meanwhile, YY1 and ZEB1 function have been related with the activation of invasion and metastasis processes (**Figure 8**). Most of the coexpression network genes carry out their function in the nucleus, some in the endoplasmic reticulum and the cytoskeleton. Additionally, the functional annotation of the coexpressed genes of the network showed an important association with cancer and the same signaling pathways of the overregulated genes in common between lung cancer and other lung diseases, with significant P values.

The library Coexnet was able to find a CCP in every comparison between the coexpression networks of the lung cancer study and the other lung diseases studies. The CCP of lung cancer and ILDs has 15 nodes and 99 edges, the CCP of lung cancer with emphysema has 185 nodes and 15,954 edges, the CCP of lung cancer and IPF has 353 nodes and 60,696 edges, and the CCP of lung cancer and PAH has 3,389 nodes and 5,537,212 edges. DAVIDs analysis of the genes in every CCP showed important associations of LC-IPF CCP with biological processes related with the acquisition of tumoral characteristics, and the LC-PAH CCP with deregulation in metabolism and signaling pathways related to inflammatory processes in cancer. The Cytoscape network analysis showed that the CCPs are highly interconnected (**Supplementary Table 3**), and highly associated with important signaling pathways deregulated in cancer, and regulated by specific TFs found in our joint transcriptomic analysis.

### Survival Analysis of Transcription Factors

**Figure 9:**
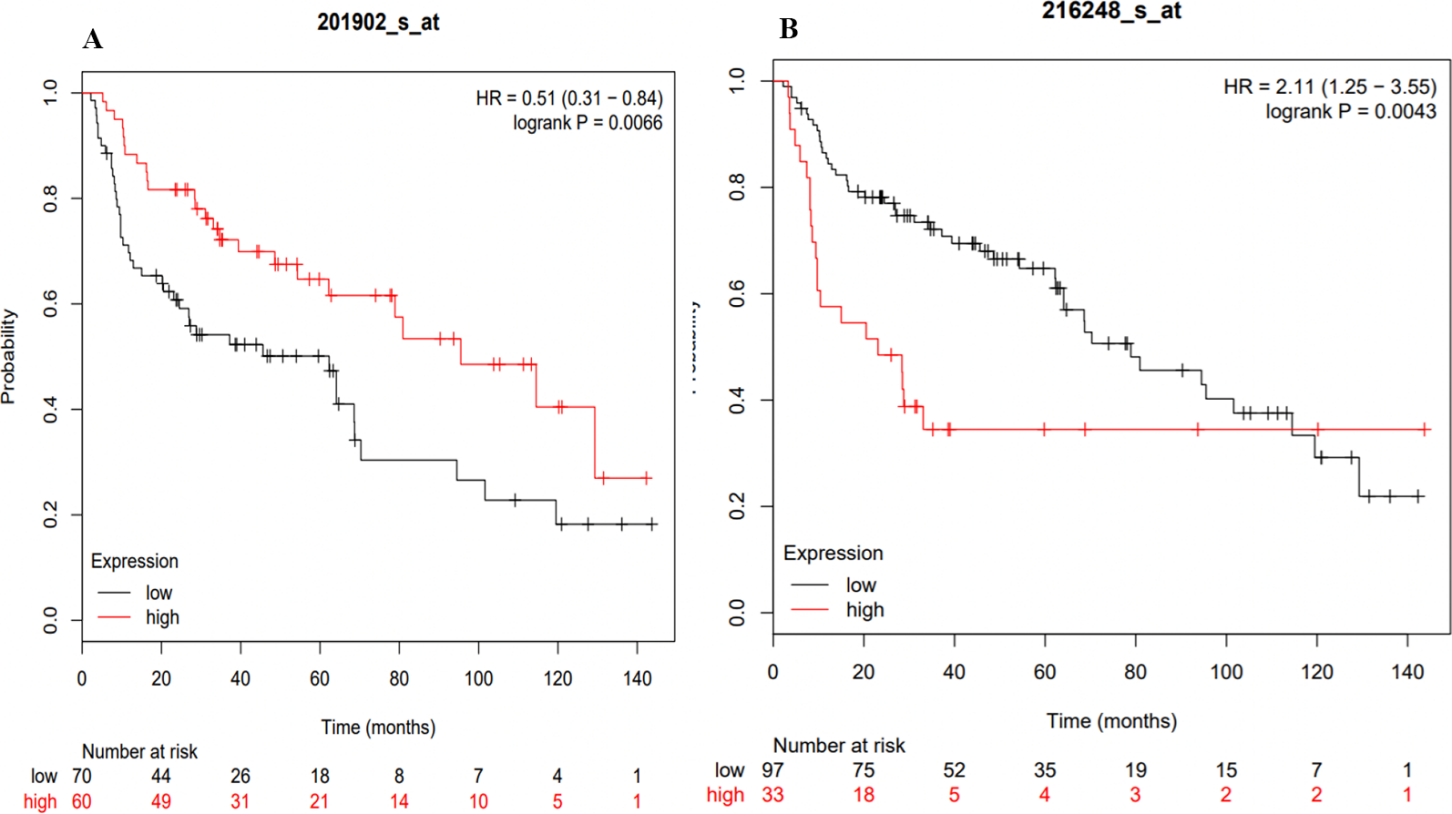
KM plots of the TFs associated with the survival of lung cancer patients. **(A)** YY1, **(B)** NR4A2.

The KM plotter analysis for the transcription factors identified during the present study revealed a statistically significant association between the expression levels of YY1 and NR4A2 with the survival of lung cancer patients (**Figure 9**) (Gyorffy et al., 2013). The multivariate analysis showed association of some of the transcription factors expression with the stage, and smoking history of lung cancer patients (**Table 2**).

**Table 2:** Kaplan–Meier multivariate analysis of transcription factors.

Variable	NR4A2		YY1	
	P value	Hazard ratio	P value	Hazard ratio
**Grade**	0.2562	0.75 (0.46–1.23)	0.7319	1.09 (0.68–1.74)
**Stage**	0.0533	0.4 (0.16–1.01)	0.1776	0.51 (0.19–1.36)
**AJCC stage T**	0.0417	1.8 (1.02–3.16)	0.0973	1.62 (0.92–2.88)
**AJCC stage N**	0.0103	3.14 (1.31–7.52)	0.0505	2.53 (1–6.44)
**Gender**	0.1274	1.52 (0.89–2.61)	0.187	1.45 (0.84–2.51)
**Smoking** **history**	0.5075	1.65 (0.37–7.34)	0.5089	1.65 (0.37–7.31)
**Survival**	0.0043	–	0.0066	–

## Discussion

Recent cancer studies have shown that it manifests as abnormal expression patterns of a significant number of genes, allowing the identification of more than 450 genetic variations through extensive GWAS association analysis (Stadler et al., 2010). The main genetic biomarkers identified have been associated with the increased risk of developing cancer (Sud et al., 2017). However, pharmaceutical gene expression regulation has not contributed significantly to stop and/or reverse tumor expansion in all patients affected with the tumoral pathology in the lung (Das et al., 2015). The great complexity of cancer etiology represented in the significant number of dysregulated genes identified, generates an intricate problem for therapeutic treatments development since it cannot be directed to each and every one of them (Gridelli et al., 2015). Moreover, until now, no biomarker has shown the ability to characterize the pathology, always reversing the tumor process in all patients affected.

Currently, neoplasms research has highlighted the ability of transcription factors (TFs) to regulate the expression of a large number of genes, which participate as important terminal regulators, being points of convergence in oncogenic signaling pathways; therefore, becoming promising biomarkers for cancer therapies (Yeh et al., 2013). TFs are important for cell differentiation during embryogenesis, but when they are deregulated in adult tissue abnormally activate signaling pathways related to tumor phenotype acquisition (Cameron and Neil, 2004). The LC&LD coexpression network analysis created with the DEGs identified with DAVIDs-Opossum analysis allowed the identification of 4 TFs capable to regulate and important number of the 35 DEGs found in common between lung cancer and other lung diseases (**Figure 5**). LC&LD network represent the genes with strongest experimental evidence about its association with tumorigenic processes, that could be associated with early stages of lung cancer, as they are also deregulated in other lung diseases. On the other hand, the CCPs found in common between lung cancer and other lung diseases, comparing lung cancer (LC) and other lung diseases (LD) coexpression networks represent another approximation that allows to find new hub genes that could be even more important for lung cancer establishment than those previously studied, because these are highly and equally connected and deregulated genes coexpressed in lung cancer and other lung diseases. For every CCP a transcription factor that regulates and important number of genes in the subnetwork was identified.

The four TFs (YY1, ZEB1, NR4A2 and E2F1) found coexpressed and identified with the DAVID-Opossum analysis have evidence of its association with tumorigenic processes, that could relate them to establishment of lung cancer. YY1 is a highly multifunctional transcription factor that promotes cell proliferation and invasion in lung cancer cells (Huang et al., 2017), and EMT in H1155 cells (Gao et al., 2018). YY1 has been associated with a regulatory loop with cancer stem cell transcription factors (SOX2, OCT4, BMI1) during the cross-talk between the NF-kB/PI3K/AKT pathways (Kaufhold et al., 2016). Therefore, YY1 overexpression in lung cancer might suggest an association of these transcription factor in driving tumorigenic processes and the acquisition of stem-like characteristics in early stages of lung cancer. YY1 is directly connected with SHMT2, a gene that support tumorigenesis (Mattaini et al., 2016), stimulates proliferation of c-Myc deficient cells (Cooper et al., 2003) and increases lung tumors growth (Zhang et al., 2012; Lee et al., 2014; DeNicola et al., 2015; Zhang et al., 2017). SHMT2 is overexpressed in all LC datasets, IPF and PAH datasets.

ZEB1 represses E-cadherin promoter, induces EMT by recruiting SMARCA4/BRG1 (Sánchez-Tilló et al., 2010), and promotes tumorigenicity by repressing stemness-inhibiting microRNAs (Wellner et al., 2009). ZEB1 expression has been related to early stage IB of NSCLC, tumor-node-metastasis stage, and EMT (Larsen et al., 2016). ZEB1 also has a tumor suppressive role, independent of its ability to induce EMT, given that ZEB1 is able to induce EMT in both KRAS- and EGFR mutant cell lines (Yochum et al., 2009). Therefore, ZEB1 downregulation in lung cancer could be related to its ability to inhibit tumor growth, which should be important to maintain in early stages of lung cancer. ZEB1 interacts with FEN1 and NR4A2, the third transcription factor coexpressed in LC&LD coexpression network. FEN1 is an enzyme that removes the 5 ‘ends during DNA repair, processes the 5’ ends of the Okazaki fragments during DNA replication (Tishkoff et al., 1997). FEN1 is an endonuclease XPG/RAD2 essential for DNA replication and the protection of the genome against mutations (Gordenin et al., 1997). FEN1 promotes tumor progression (He et al., 2017a), proliferation and poor prognosis of NSCLC (Zhang et al., 2018a). FEN1 is overexpressed in all LC datasets and PAH dataset. YY1 and ZEB1 also appeared as the regulators of the CCP formed between LC and Emphysema, giving another layer of evidence of their importance in the establishment of LC in patients with an emphysema antecedent.

NR4A2 transcriptionally regulates cell proliferation, apoptosis, inflammation, neuronal development, and carcinogenesis (Safe et al., 2013; Safe et al., 2016). NR4A2 overexpression blocks p53 target genes activation, like mir-34a, which rescues cells from p53-induced inhibition of proliferation (Beard et al., 2016). NR4A2 is downregulated in lung cancer and overexpressed in PAH dataset, suggesting an association with tumoral processes during early stages of lung cancer related to this lung disease. NR4A2 only interacts with ZEB1, the second transcription coexpressed in the network, which might suggest a possible coregulatory relationship between these two transcription factors and the association with the same tumorigenic processes related with ZEB1.

E2F1 is a transcription activator overexpressed in lung adenocarcinoma and squamous cell lung carcinoma tissues, associated with cellular proliferation by counteracting the negative effects of cyclin-cdk inhibitors (Eymin et al., 2001). ANKRD22 promotes progression of NSCLC through transcriptional up-regulation of E2F1 (Yin et al., 2017). E2F1 was one the transcription factor that Opossum selected as possible regulator of the 45 common genes overregulated and related to cancer, and appeared in the coexpression network, even when it is not deregulated in the selected sets of other lung diseases, probably suggesting its importance at the end of early stages of lung cancer. Moreover, E2F1 was the TF identified as the most important regulator with iRegulon for the LC&LD coexpression network, in terms of enrichment if its TFBS, and the number of network targets that can regulate. E2F1 interacts with SHMT2 like YY1, and RAD54L. RAD54L is a recombinational DNA repair protein, and a double-stranded DNA-dependent ATPase that stimulates homologous recombination, inducing a DNA topological changes and induces DNA repair of DNA double-strand breaks (Swagemakers et al., 1998; Ristic et al., 2002; Sigurdsson et al., 2002). RAD54L is upregulated in NSCLC and associated with dsDNA break repair (Välk et al., 2011). RAD54L is overexpressed in all lung cancer datasets and PAH dataset.

The CCP formed between LC and ILD is regulated by IRF1, a transcriptional regulator of cellular responses, during hematopoiesis, inflammation, immune responses, cell proliferation and differentiation (Romeo et al., 2002). IRF1 regulation is associated with cell cycle, induction of growth arrest and programmed cell death following DNA damage (Bowie et al., 2008). IRF1 is downregulated in lung cancer, IPF and PAH datasets, probably because it represses the expression of genes involved in anti-proliferative response, such as BIRC5/survivin, CCNB1, CCNE1, CDK1, CDK2 and CDK4 (Armstrong et al., 2012) and in immune response, such as FOXP3, IL4, ANXA2 and TLR4 (Fragale et al., 2014). IRF1 stimulates p53/TP53-dependent transcription, improving EP300 recruitment which leads to an increase of p53/TP53 acetylation (Dornan et al., 2004). IRF1 is also known as tumor suppressor, capable to avoid tumor cell growth (Bouker et al., 2005), and stimulate an immune response against tumor cells (Tanaka et al., 1996).

The CCP formed between LC and IPF is regulated by NF1, a Ras‐GTPase activating protein, which function as negative regulators for Ras proteins (Ballester et al., 1990), preventing the downstream activation of Ras effector pathways, MAPK and PI3K/Akt/mTOR pathways that drive the pro-proliferation, survival, differentiation (Xu et al., 1990; Bollag and McCormick, 1991), cell adhesion, and migration (Kweh et al., 2009). NF1 is a mutated tumor suppressor in both of the NSCLC subtypes, adenocarcinoma (Collisson et al., 2014) and squamous cell (Hammerman et al., 2012). Lung cancers with NF1 mutations have also been characterized by downstream activation of Ras signaling (Redig et al., 2016). Therefore, there is evidence that suggest that NF1 mutations in lung cancer con also activate Ras signaling pathway, playing a key role in the incorrect signal transduction, proliferation and malignant transformation (Reuter et al., 2000). The CCP formed between LC and PAH is regulated by BRCA1, a E3 ubiquitin-protein ligase that mediates ‘Lys-6’-linked polyubiquitin chains formation, enabling cellular responses to DNA damage during DNA repair (Lee et al., 2000; Wang et al., 2007). BRCA1 has a tumor suppressor function related to the transcriptional regulation to maintain genomic stability, which is directly related to hereditary breast cancer (Li and Greenberg, 2012; Sedic et al., 2015). However, BRCA1 is overregulated in LC and PAH, and it expression has been related to the occurrence and development processes of NSCLC (Sun et al., 2018b).

The probability of surviving was associated with the expression of two transcription factors (YY1 and NR4A2) (**Figure 9**) identified with the coexpression analysis. Moreover, the Kaplan–Meier Multivariate analysis also associate their expression with stage, gender and smoking history. Therefore, the clinical history of patients could help in the future to prevent or apply most accurate treatments to lung cancer patients. At the moment, the clinical data highlights that risk of developing lung cancer is 1.5 times higher in patients with ILD compared with COPD and with the general population (Jung et al., 2011), while lung cancer prevalence in IPF patients can reach 48% (Le Jeune et al., 2007).

Most of the genes in the LC&LD coexpression network are deregulated in PAH and some in IPF, and only one in ILDs and Emphysema (**Supplementary Table 2**). The pathogenesis of PAH has been associated with genetic factors that confer a predisposition for development or progression, such as mutations of the bone morphogenic receptor type II gene (BMPR2), a member of the transforming growth factor superfamily TGF-β (Newman et al., 2001), mutations in the activin A receptor type II 1 (ACVRL1) (Girerd et al., 2010), endoglin (ENG) (Chaouat et al., 2004), SMAD9, caveolin-1 (CAV1) and KCNK3 (Ma et al., 2013). The majority of BMPR2 mutations result in a loss of function, but show low penetrance (∼20%) in more than 70% of patients with familial PAH and in 11–40% of patients with sporadic PAH, affecting only 20% of carriers during their lifetime (Lane et al., 2000; Machado et al., 2001). However, BMPR2 is one of the “guardians” of pulmonary vessels homeostasis during repair processes, controlling both apoptosis and cell proliferation of pulmonary vascular smooth muscle and endothelial cells (Morrell, 2006). Therefore, epigenetic studies have begun to study the regulatory mechanisms associated with BMP signaling in abnormal BMPR2 function, without necessarily implying a mutation in the BMPR2 gene, such as, for example, the importance of BMP/TGF-β signaling in maintaining normal pulmonary arteriolar structure (Trembath et al., 2001; Nasim et al., 2011), STAT3/miRNA-17-92 axis activation in normal human lung endothelial cells after interleukin (IL) -6 exposure (Brock et al., 2009), leads to the development of PAH (Steiner et al., 2009), and angiobliterant vascular remodeling and robust hypertrophy of the right ventricle (Gomez-Arroyo et al., 2012). Overexpression of miR-17 increases the proliferation of smooth muscle cells of human pulmonary arteries, and its inhibition improves pulmonary and cardiac function (Pullamsetti et al., 2012). The miR-204 is negatively regulated in the smooth muscle cells of the pulmonary artery isolated from patients with PAH, and an apoptosis-resistant phenotype in smooth muscle cells is associated with induction (Courboulin et al., 2011).

Patients with pulmonary arterial hypertension (PAH) have a sustained elevation of resistance and pulmonary arterial pressure, which makes them increasingly narrow until they are completely blocked (Montani et al., 2013), leading to the pumping of blood by the heart weakens the muscles until they reach heart failure through the right ventricle (Bogaard et al., 2009). Likewise, PAH is characterized by vascular remodeling in the three layers (intimate, middle and adventitia) of the wall of small and medium pulmonary arterioles (<500 µm), in response to continuous vascular lesions that lead to abnormal muscle muscularization of distal and medial capillary arteries, loss of precapillary arteries, and thickening of arterial walls (Guignabert and Dorfmuller, 2013). PAH begins to develop under constant stress conditions such as inflammation and pseudo-hypoxia, initially inducing the death of endothelial cells (Guignabert and Dorfmuller, 2013). However, cells eventually acquire hyperproliferative abilities (Lee et al., 1998), associated with instability of short sequences of microsatellite DNA within plexiform lesions (Yeager et al., 2001), and the presence of somatic chromosomal abnormalities in the lung patients with PAH (Aldred et al., 2010); a deregulated cell metabolism (Xu et al., 2007; Aldred et al., 2010; Sutendra et al., 2010; Tuder et al., 2012), with reduced oxygen consumption, reduced mitochondrial respiration and increased glycolytic metabolism (Xu et al., 2007); and the ability to evade apoptosis (Masri et al., 2007), with an increase in the expression of important anti-apoptotic genes such as Bcl-xL, Bcl-2 and survivin (McMurtry et al., 2005; Tu et al., 2012); in clones of fibroblasts, smooth muscle cells (PASMC) and endothelial cells of the pulmonary arteries (PAEC), which allows vascular remodeling during PAH (Guignabert et al., 2013).

The hypothesis of establishment or “quasi-malignancy” is also associated with the concept of an angiogenic/stem cell tumor (CSC) niche (Rai et al., 2008). Precursor cells can divide and migrate to pulmonary vascular adventitia through the vasa vasorum (Ergün et al., 2011; Yeager et al., 2011), and stem cells of small microvascular pulmonary endothelial cells of small arteries can proliferate to the point of obliteration (Alvarez et al., 2008). The pathobiological model of intraluminal angioproliferation is based on the growth of endothelial cells in PAH, where the “endothelial cell monolayer law” has been broken in favor of a tumor-like intraluminal growth (Tuder et al., 2001; Sakao et al., 2005). The endothelial cell such as the cell that initiates a complex lesion can come from three possible sources of proliferating endothelial cells: (1) An endothelial cell similar to a stem cell that is resistant to apoptosis and grows after neighboring monolayer cells intimate have been injured and have died (Yoder et al., 2007), (2) A precursor cell derived from the bone marrow (Sahara et al., 2007), and (3) An endothelial cell derived from the vascular endothelial growth factor (VEGF) action in dendritic cell-directed transdifferentiation (Steinman and Banchereau, 2007). The complex vascular lesions of PAH are governed by some, but not all, cancer characteristics. Therefore, although the lesions are not completely tumor, they are certainly neoplasms in the sense that there is a process of abnormal and uncontrolled cell growth.

Pulmonary arterial hypertension (PAH) showed great complexity as did cancer, something that could be evidenced by the large number of DEGs, comparable to those observed in lung cancer, which may be partly explained by PAH is so difficult to treat. Vasodilator-based treatments have not demonstrated the ability to reverse vascular remodeling and do not affect survival (Lajoie et al., 2016), but the acquisition of cancer characteristics in PAH-associated cells suggests the potential use of anticancer drugs in patients with PAH, such as imatinib, a tyrosine kinase inhibitor, which could potentially be beneficial in patients with PAH after evaluation of potential side effects for safe use in clinical practice (Hoeper et al., 2013).

## Conclusion

In the present work, we show how the re-analysis of gene expression data available in the databases within an organized and focused bioinformatic pipeline can be used to obtain new knowledge and give more evidence that supports a biological hypothesis, as well as find potential biomarkers associated with molecular functions in common for different diseases. The methodology we propose for the identification of the transcriptional regulators of all deregulated genes related to the acquisition of the hallmarks of cancer during lung tumorogénesis, allowed us to truly visualize the massive complexity of lung cancer. The transcriptomic analyzes of multiple gene expression data and the creation and comparison of coexpression networks allowed to identify all the genes involved and the molecular events associated with the lung tumoral process, and the transcription factors or regulators of all these genes that can participate in the establishment of the disease, as biomarkers, which will lead in the future to establish in each patient the specific molecular event that causes the pathology, and to implement an effective personalized therapy against lung cancer.

The existence of common DEGs among different lung pathologies strengthens the experimental evidence that supports the hypothesis about a possible causal relationship between different lung diseases and lung cancer, because the deregulated biological functions in other lung diseases are related with oncogenic processes, and may be the first functions affected and those necessary for the establishment of a tumor pathology. Therefore, this group of genes can be more specific for the early stages of lung cancer, and might be more useful for early detection, and eventually could be integrated into the clinical practice to increase early detection probability of lung cancer, and used in the development of personalized therapies. The TFs coexpressed in the LC&LD network and the CCPs LC–LD have experimental evidence of their association with the acquisition of hallmarks of cancer during its establishment that must be validated experimentally. Common DEGs and TFs among lung pathologies might have the potential to be used as possible biomarkers for diagnosis and treatment that should be evaluated experimentally in patients, to demonstrate that its deregulation is crucial for the initiation and establishment of the tumor process in the lung, and therefore, the regulation of its expression is sufficient to prevent, control and revert the tumor process in a more efficient and specific way in every patient.

The levels of expression of the TFs associated with the establishment of lung according to our joint transcriptomic and coexpression analysis must be evaluated experimentally, along with the expression of the winner DEGs coexpressed in lung cancer. Functional analysis must be performed for every TF and winner DEGs to establish the association with the acquisition of which hallmark of cancer are related. Moreover, all functional and direct targets of every TF identified in lung cancer must be identified to fully understand their regulatory function during early stages of the disease. Also, the TFs identified as the key regulators for its ability to control and reverse the tumor process must be studied in depth, to find the regulatory mechanisms associated with their expression in lung cancer, like histone modifications, DNA methylation and non-coding RNAs, as the basis to develop specific and effective techniques of early detection and treatments against the tumoral pathology of the lung.

The joint transcriptomic laid the foundation to guide the research field of our lab providing potential candidate biomarkers that must be experimentally validated within the all regulatory transcriptional network of lung cancer, to become prognostic, diagnostic, and predictor biomarkers of lung cancer, which in the future will also guide the clinician process to establish the specific molecular event that causes a tumoral pathology in each patient, and to implement an effective personalized therapy against the disease.

## Data Availability Statement

All microarray data sets are available for the scientific community, in the Gene Expression Omnibus (GEO) and ArrayExpress.

## Author Contributions

LL-K proposed the combined analysis to identify common genes between pathologies, the step-by-step methodology applied and led the process of data analysis. MF did the data pre-processing and the detection of differentially expressed genes (DEGs) using specific libraries in R. BO-O did the functional/enrichment analysis, the literature searching, the coexpression network and survival analysis, and contribute with the manuscript writing. DU contributed with the literature searching of other lung diseases. AR and AA contributed to the study conception and design, leading the results interpretation and making important contributions to the *Discussion* section. All authors critically reviewed and approved final manuscript and agree to be accountable for all aspects of the work.

## Funding

The current article was funded by the grant number 41492 from Universidad Nacional de Colombia, and PUJ ID7687 from Pontificia Universidad Javeriana.

## Conflict of Interest

The authors declare that the research was conducted in the absence of any commercial or financial relationships that could be construed as a potential conflict of interest.
