# Supplementary material for: Joint Transcriptomic Analysis of Lung Cancer and Other Lung Diseases
Source: Front Genet. 2019 Dec 6;10:1260. doi: 10.3389/fgene.2019.01260 (PMC6908522; doi:10.3389/fgene.2019.01260)
Supplement: Supplementary file 2 [file Table_2.docx]

**Supplementary Table 2** Biological function and experimental evidence of the LC&LD coexpression network genes.

| Gene | Molecular function | Biological process | LC&LD | LD dataset |
| --- | --- | --- | --- | --- |
| ASCC3 | Helicase, Hydrolase, RNA binding | DNA damage, DNA repair, Transcription regulation, cell proliferation, DNA repair, DNA duplex unwinding. | Overexpression associated with lung fibrosis and cancer (Gahring et al., 2017). Deregulation associated to COPD (Sauler et al., 2018). | Overexpressed in IPF dataset. Downregulated in PAH (5:2). |
| ATP2A2 | Translocase | Calcium transport, Ion transport, Transport | Related to colon and lung cancer early events (Korošec et al., 2006). Associated with COPD (Wang et al., 2008). Deregulated un pulmonary fibrosis (Patel et al., 2013). | Overexpressed in IPF dataset. Downregulated in PAH dataset (5:2). |
| AURKB | Kinase, Serine/threonine-protein kinase, Transferase | Cell cycle, Cell division, Mitosis | A potential target in NSCLC progressing to anti-EGFR therapy (Bertran-Alamillo et al., 2019). Inhibition therapy can specifically target KRAS-transformed cells (Dos Santos et al., 2016). | Overexpressed in IPF dataset. Downregulated in PAH dataset (5:2). |
| BIRC5 | Protease inhibitor, Repressor, Thiol protease inhibitor | Apoptosis, Cell cycle, Cell division, Chromosome partition, Mitosis, Transcription, Transcription regulation | Upregulation in cell cycle signaling pathway in LAD (Cao et al., 2019), to a history of smoking in LAD (Hirano et al., 2015), to early-stage of NSCLC (Falleni et al., 2003), and PAH (Blanco et al., 2016). | Overexpressed in PAH dataset (6:1). |
| BUB1B | Kinase, Serine/threonine-protein kinase, Transferase | Apoptosis, Cell cycle, Cell division, Mitosis | A critical role in poor prognosis and tumor progression of lung adenocarcinoma (Chen et al., 2015). Upregulated in peripheral blood mononuclear in IPF (El-Chemaly et al., 2018a). | Overexpressed in PAH dataset (6:1).. |
| CCNA2 | Cyclin | Cell cycle, Cell division, Host-virus interaction, Mitosis | Facilitates EMT via the integrin αvβ3 signaling in NSCLC (Shan Ruan et al., 2017). Related to IPF (Vukmirovic and Kaminski, 2018) and apoptosis (Konishi et al., 2009). | Overexpressed in IPF and PAH datasets (6:2) |
| CCNB1 | Cyclin | Cell cycle, Cell division, Mitosis | A prognostic marker of early-stage SCC of the lung (Soria et al., 2000). Upregulated in both HPAH and IPAH (Yu et al., 2015). | Overexpressed in IPF dataset (6:1) |
| CCNB2 | Cyclin | Cell cycle, Cell division, Mitosis | Increased in IPAH, and HPAH (Yu et al., 2015). Upregulation involved in sepsis-related acute respiratory distress syndrome (Wang et al., 2016). | Overexpressed in PAH dataset (6:1). |
| CENPE | Developmental protein, Motor protein | Cell cycle, Cell division, Mitosis | Promotes proliferation of LUAD cells (Shan et al., 2019). Maintains chromosomal stability (Putkey et al., 2002). | Overexpressed in PAH dataset (6:1). |
| CHAF1A | Chaperone | Cell cycle, DNA damage, DNA repair, DNA replication | Associated with proliferation, invasion and migration of lung cancer cells (Cai et al., 2018). Upregulated in NSCLC, promoting proliferation and colony formation (Liu et al., 2017a). | Overexpressed in PAH dataset (6:1). |
| COL1A1 | Extracellular matrix structural constituent | Cellular response to multiple stimuli | Overexpression in NSCLC under stress conditions (Oleksiewicz et al., 2017). Associated with Emphysema (PIERCE et al., 1961), and IPF (Yang et al., 2013). | Overexpressed in Emphysema dataset (6:1). |
| DTL | Substrate-specific adapter of a DCX (DDB1-CUL4-X-box) E3 ubiquitin-protein ligase complex | Biological rhythms, DNA damage, DNA replication, Ubl conjugation pathway | Correlated with overall survival, with cell cycle and DNA repair pathways in LuADCs (Yan et al., 2017). miR-30a regulates directly DTL in cell proliferation (Jiang et al., 2018). | Overexpressed in PAH dataset (6:1). |
| E2F1 | Activator, DNA-binding | Apoptosis, Cell cycle, Transcription, Transcription regulation | Higher expression in LAD and SCL (Sun et al., 2018a). Associated with the proliferation of NSCLC cells (Yu et al., 2017). | Overexpressed only in LC datasets (5:0). |
| FEN1 | Endonuclease, Exonuclease, Hydrolase, Nuclease | DNA damage, DNA repair, DNA replication | Associated with the initiation of NSCLC (Zheng et al., 2007a). Promotes tumor progression in NSCLC (He et al., 2017a), proliferation and poor prognosis of NSCLC (Zhang et al., 2018a). | Overexpressed in PAH dataset (6:1). |
| HMMR | Hyaluronic acid binding | Hyaluronan catabolic process Source: Reactome, Regulation of G2/M transition of mitotic cell cycle. | Knockdown of HMMR in LUAD cells decreased their potential to invade, migrate, and form colonies in vitro (Stevens et al., 2015, 2017). | Overexpressed in PAH dataset (6:1). |
| KIF11 | Motor protein | Cell cycle, Cell division, Mitosis | Overexpression in NSCLC (Schneider et al., 2017a), and tumor growth (Kato et al., 2018). Overregulated in PBMC FPF (El-Chemaly et al., 2018b), in HPAH and IPAH (Yu et al., 2015). | Overexpressed in PAH dataset (6:1). |
| KIF2C | ATPase activity, centromeric DNA binding, Central microtubule motor activity | Cell cycle, Cell division, Chromosome partition, Mitosis | Overexpressed in cancer (Huang and Gao, 2018; Li et al., 2018; Song et al., 2018). Association with progression and prognosis of LUAD (Bai et al., 2019). | Overexpressed in IPF and PAH datasets (6:2) |
| MCM2 | DNA-binding, Helicase, Hydrolase | Cell cycle, DNA replication | Involved in lung cancer cell proliferation, cell cycle, and migration (Cheung et al., 2017). Prognostic significance in NSCLC (Yang et al., 2006). A marker of premalignant lung cells (Tan et al., 2001). | Overexpressed in PAH dataset (6:1). |
| MCM4 | DNA-binding, Helicase, Hydrolase | Cell cycle, DNA replication | Role in the proliferation of NSCLC cells. High level of MCM4 expression (>70%) had significantly shorter survival in the adenocarcinoma group (Choy et al., 2016). | Overexpressed in IPF dataset (6:1) |
| MCM6 | DNA-binding, Helicase, Hydrolase | Cell cycle, DNA replication | Overexpression in NSCLC is associated with poor prognosis (Liu et al., 2017b; Vigouroux et al., 2015). A diagnostic biomarker for lung cancer early detection (Liu et al., 2014). | Overexpressed in PAH and IPF datasets (6:1). |
| MCM7 | DNA-binding, Helicase, Hydrolase | Cell cycle, DNA replication | Related to cell cycle progression in NSCLC (Fei et al., 2017). Overexpression in poorly differentiated tumors and non-bronchioloalveolar carcinomas (Liu et al., 2012). | Overexpressed in PAH dataset (6:1). |
| MKI67 | DNA-binding, RNA binding | Cell cycle | Proliferation index (overall mean: 40.7%) differed significantly according to histologic subtypes with SQCC showing a mean PI (52.8%) twice as high as ADC (25.8%) (Warth et al., 2014). | Overexpressed in PAH dataset (6:1). |
| MRPS30 | Ribonucleoprotein, Ribosomal protein | Apoptotic process, mitochondrial translational elongation, mitochondrial translational termination | Mitochondrial markers predict survival and progression in non-small cell lung cancer (NSCLC) patients (Sotgia and Lisanti, 2017). | Overexpressed in PAH dataset (6:1). |
| MSH2 | DNA-binding | DNA damage, DNA repair | MSH2 is a tumor suppressor gene. The reduction in expression is a late event in the development of invasive malignancy and does not influence survival of NSCLC (Cooper et al., 2008). | Overexpressed in PAH dataset (6:1). |
| NR4A2 | DNA-binding, Receptor | Transcription, Transcription regulation | Regulates cell proliferation, apoptosis, inflammation, neuronal development, and carcinogenesis (Safe et al., 2013, 2016). Overexpression blocks p53 target genes (Beard et al., 2016). | Overexpressed in PAH dataset (5:1). |
| PARP1 | DNA-binding, Glycosyltransferase, Transferase | DNA damage, DNA repair, Transcription, Transcription regulation | Associated with the metastatic potential of lung adenocarcinoma cells (Choi et al., 2016). | Overexpressed in PAH dataset (6:1). |
| PCNA | DNA-binding, DNA polymerase binding, enzyme binding, estrogen receptor binding | DNA damage, DNA repair, DNA replication, Host-virus interaction | Overexpression associated with NSCLC proliferation rate (Stoimenov and Helleday, 2009), behavior (Zhu et al., 2010). Overexpression in IPF (Chhina et al., 2010; Kogan et al., 2010). | Overexpressed in IPF dataset (6:1). |
| RAD54L | DNA-binding, Helicase, Hydrolase | DNA damage, DNA repair | Upregulated in NSCLC (Välk et al., 2011). RAD54L and RAD54B counteract genome-destabilizing effects of direct binding of RAD51 to dsDNA in human tumor cells (Mason et al., 2015). | Overexpressed in PAH dataset (6:1). |
| RFC2 | ATP binding, DNA binding, enzyme binding | DNA replication | Tumor progression determinants of NSCLC: PRPF19, MCM4, MCM5, RFC2, PLK1, CDK2, PDGFB, E2F (Relli et al., 2018). | Overexpressed in PAH dataset (6:1). |
| RFC3 | DNA-binding, DNA clamp loader activity | DNA replication | Overregulation involved in cancer cell proliferation, tumor volume, weight and relieve lung metastasis in vivo (He et al., 2017b). | Overexpressed in PAH dataset (6:1). |
| RRM1 | Allosteric enzyme, Oxidoreductase | DNA replication | Overexpression in NSCLC and treated with chemotherapy (Bepler et al., 2006; Carvalho et al., 2009). Determinant of survival after surgical treatment of early-stage NSCLC (Zheng et al., 2007b). | Overexpressed in PAH dataset (6:1). |
| RSRC1 | Spliceosome assembly | mRNA processing, constitutive and alternative mRNA splicing | Important role in neural stem cell proliferation, cancer development, and cell growth inhibition in neuroblastoma cells (McDaniel et al., 2017). | Overexpressed in PAH dataset (6:1). |
| SCG5 | Chaperone, Neuropeptide, enzyme inhibitor activity, unfolded protein binding | Transport | Overexpressed in SCLC plasma (Duijnhoven et al., 1991; Iguchi et al., 1989; Roebroek et al., 1989). SCG5 over-expression correlation with ILDs gene expression (Christmann et al., 2016). | Overexpressed in IPF dataset. Downregulated in PAH dataset (5:2). |
| SHMT2 | Transferase | One-carbon metabolism | Overexpression supports tumorigenesis (Mattaini et al., 2016), proliferation (Cooper et al., 2003) and increase lung tumors growth (DeNicola et al., 2015; Lee et al., 2014; Zhang et al., 2012, 2017). | Overexpressed in IPF and PAH datasets (6:2) |
| SPP1 | Hydrolase | ER to Golgi ceramide transport, extrinsic and intrinsic apoptotic signaling pathway, sphingosine metabolic process | Promotes lung tumor dissemination (Giopanou et al., 2016), reduces survival of LUAD patients (Zhang et al., 2018c). A marker of disease severity in PAH (Mura et al., 2019). | Overexpressed in IPF dataset. Downregulated in PAH dataset (5:2). |
| TOP2A | DNA-binding, Isomerase, Topoisomerase | Biological rhythms | Correlated to LAD worse survival (Dingemans et al., 2001; Hou et al., 2017). A proliferation marker that could distinguish bronchopulmonary neuroendocrine neoplasms entities (Neubauer et al., 2016). | Overexpressed in PAH dataset (6:1). |
| TOPBP1 | DNA-binding | DNA damage, DNA repair | Induction of E2F1-dependent apoptosis upon depletion of TopBP1 (Liu et al., 2004; Yoshida and Inoue, 2004). Keeps p53 activities in check during normal growth. (Liu et al., 2009). | Overexpressed in PAH dataset (6:1). |
| TPX2 | protein kinase binding | Apoptosis, Cell cycle, Cell division, Mitosis | Overexpress in NSCLC (Schneider et al., 2017b). Associated with poor prognosis of smoking related lung adenocarcinoma (Zhang et al., 2018b). | Overexpressed in PAH dataset (6:1). |
| YY1 | Activator, DNA-binding, Repressor | Differentiation, DNA damage, DNA recombination, DNA repair, Spermatogenesis, Transcription regulation | Ectopic expression in lung cancer cells promote cell proliferation and invasion (Huang et al., 2017), and EMT (Gao et al., 2018). | Downregulated in PAH dataset (3:1). |
| ZEB1 | Activator, DNA-binding, Repressor | Differentiation, Neurogenesis, Transcription, Transcription regulation | Required in EMT, promotes malignant progression, tumorigenicity, invasion, and metastases in NSCLC lines (Larsen et al., 2016), through NOTCH1 (Zhang et al., 2016a). | Overexpressed in PAH dataset. Downregulated in ILDs (4:2). |
| ZWILCH | Kinetochores assemble, proteinaceous multi-subunit assembly | Cell cycle, Cell division, Mitosis | High expression correlated with increased levels of genomic instability and adverse tumor properties, poor lung cancer patients survival, especially early-stage tumors (Zhang et al., 2016b). | Overexpressed in PAH dataset (6:1). |
